# 

**Published:** 1916-10

**Authors:** 


### Contents

Event and Comment .... 451

The Prevalence of Chronic Mouth Infections and their Management .... 453

The Principles Involved in Focal Infection as Related to Systemic Disease .... 461

Dental Infections and Systemic Disease—Treatment and Results .... 472

Abstract of Discussion .... 481

Stop! .... 487

The Editor's Desk .... 489

Indents .... 491

Announcements .... 497

